# Cationic gas-filled microbubbles for ultrasound-based nucleic acids delivery

**DOI:** 10.1042/BSR20160619

**Published:** 2017-12-22

**Authors:** Anthony Delalande, Colette Bastié, Lucie Pigeon, Simona Manta, Matthias Lebertre, Nathalie Mignet, Patrick Midoux, Chantal Pichon

**Affiliations:** 1 Centre de Biophysique Moléculaire CNRS UPR 4301, rue Charles Sadron, Orléans 45071, France; 2 Génétique Immunothérapies Chimie et Cancer CNRS UMR 7292, 10 Boulevard Tonnellé BP 3223, Tours 37032, Cedex 01, France; 3 Unité des Technologies Chimiques et Biologiques pour la Santé. Equipe Vecteurs pour l’Imagerie Moléculaire et la Thérapie Ciblée CNRS UMR8258, INSERM U1022, ENSCP Chimie ParisTech, 4 avenue de l’Observatoire, Paris 75006, France; 4 Transderma Systems, 31 Avenue Monge, Tours 37200, France

**Keywords:** cationic, gene delivery, microbubble, ultrasound

## Abstract

The use of ultrasound has gained great interest for nucleic acids delivery. Ultrasound can reach deep tissues in non-invasive manner. The process of sonoporation is based on the use of low-frequency ultrasound combined with gas-filled microbubbles (MBs) allowing an improved delivery of molecules including nucleic acids in the insonified tissue. For *in vivo* gene transfer, the engineering of cationic MBs is essential for creating strong electrostatic interactions between MBs and nucleic acids leading to their protection against nucleases degradation and high concentration within the target tissue. Cationic MBs must be stable enough to withstand nucleic acids interaction, have a good size distribution for *in vivo* administration, and enough acoustic activity to be detected by echography. This review aims to summarize the basic principles of ultrasound-based delivery and new knowledge acquired in these recent years about this method. A focus is made on gene delivery by discussing reported studies made with cationic MBs including ours. They have the ability for efficient delivery of plasmid DNA (pDNA), mRNA or siRNA. Last, we discuss about the key challenges that have to be faced for a fine use of this delivery system.

## Introduction

During these last two decades, chemical and physical methods have been developed at an amazing speed to improve drug/gene delivery. It is admitted that designing an efficient, targeted delivery system is the ultimate step to further improve delivery efficacy and reduce the side effects as much as possible due to non-specific activity. In this respect, strategies that could selectively target tissues of interest are still an ongoing story both in experimental and clinical research areas. Ultrasound was first applied in the medical field for imaging as ultrasonography and for physical therapy based on pulsed ultrasound mode [1,2]. Last, therapeutic applications of ultrasound have gained new interests as a result of its use as an external trigger for drug or gene delivery. Ultrasound waves enable to spatiotemporally control the release in a non-invasive manner of a drug encapsulated in gas-filled microbubbles (MB) or in their surroundings [3–7]. Depending on the acoustic energy applied, thermal or non-thermal effects can be produced by ultrasound, each of them has their own application. High ultrasound intensities produce heating due to the absorption of acoustic energy by tissues. This property is employed in high-intensity focused ultrasound (HIFU) surgery [8] or ultrasound-based physiotherapy [9]. It has been stated that an elevation of 1.5°C during 5 min is considered safe, while an elevation of 4–5°C could be damaging for tissues [10]. At low ultrasound intensities, cavitation, mechanical streaming, radiation forces and shock waves are the main non-thermal effects obtained. These effects may result into some benefits such as tissue healing for bone [11], cartilage [12] or tendon [13]. Another effect is the improvement of gene or drug delivery, thanks to ultrasound trigger assisted by MB. Mechanical streaming and radiation forces induced by MB are effective to enhance the diffusion of drugs across the vessel wall [14,15]. These effects have been widely studied to open transiently the blood–brain barrier [16] or blood–tumor barrier [17]. Inertial cavitation is the process of formation, oscillation and collapse of gaseous bubbles driven by an acoustic field. The presence of preformed MBs in the environment allows reducing the threshold of energy needed for cavitation decreasing the harmful effects.

## Ultrasound contrast agents: gas-filled MBs

MBs are gas-filled vesicles or particles consisting of a gas core encapsulated by a stabilizing shell. They had been first developed as contrast agents for ultrasound imaging. When injected intravenously, these MBs remain within the vasculature where they increase the blood echogenicity. In this way, enhanced contrast between blood and soft tissues can be achieved to differentiate blood from their surroundings under ultrasound due to their high acoustic impedance mismatch [18]. Most of FDA and EMEA contrast agents such as Optison™, Definity™, Sonazoid™ and SonoVue™ for echography are lipid-based MBs allowing good oscillations when driven by ultrasound. Specific non-linear oscillations can be detected in ultrasound contrast imaging based on MB harmonics detection [19]. Another application of these MB concerns their ability to improve imaging of lymphatic vessels for sentinel lymph nodes detection. In oncology, the sentinel lymph node detection and resection are standard procedures to remove metastatic tumor cells. The detection of the proximal lymph node of the tumor is of importance since this node would be the first roundabout for metastasis of tumor cells. Once injected intradermally or subcutaneously, MBs could be drained into the lymphatics and reach the lymph nodes [20]*.* This observation led to their exploitation for cancer imaging as a novel sentinel lymph node detection, which is non-radioactive and non-invasive [21,22]. In addition to their role in diagnostic ultrasound imaging after systemic injection, MBs also function as a nuclei for acoustic cavitation in ultrasound-mediated gene delivery [23]. MBs are very effective ultrasound scatters due to their high compressibility. Upon insonification, they start oscillating at the frequency of ultrasound, under the influence of positive and negative pressure differences in the ultrasonic wave [24,25]. Due to their acoustic behaviour, MBs can induce an increased surrounding cells permeability allowing drug delivery [26,27]. The increased uptake by ultrasound in presence of MBs has been attributed to the formation of transient pores in the plasma membrane with a phenomenon called ‘sonoporation’. The acoustic response of MBs depends on the intensity of ultrasound wave applied and the type of MB’s shell. MBs have a biodegradable shell that can be composed of lipids, proteins, sugars or polymers and they have a heavy gas core instead of air to provide additional MB stability [26]. The low diffusion rate of high molecular weight perfluorocarbons prolongs their stability [28].

During storage and *in vivo* circulation, MB stability is extremely important to consider. It depends on the shell and the gas composition. The elasticity and the thickness of the shell are important parameters. MBs having a very soft shell would be disrupted at small pressure variations. On the opposite, MBs with a hard shell would be less able to oscillate. A good compromise must therefore be found. The most elastic shells are made of phospholipids, while the stiffer ones are made of polymers or proteins. Customized MBs are mainly made with classical phospholipids, PEGylated lipids and perfluorocarbon gas. The hydrophobic tails of the lipids associate with the gas phase, and the hydrophilic head groups interact with the water molecules. The size of MB also determines their stability. Larger the MBs the more they are stable, but for intravenous administration the MB diameter must be smaller than 6 µm [29]. The MB shell architecture and its composition have a profound impact on their response in an ultrasonic field, which potentially can reduce transfection efficiencies [30,31]. Standard production techniques are based on emulsion by shaking or sonication. They result in MBs with a wide size distribution. For therapeutic applications, a high level of control of their size and uniformity is required in particular. The broader the MB size distribution is, the smaller will be the proportion of MB excited at maximum amplitude. Some novel preparation techniques such as coaxial electrohydrodynamic atomization (CEHDA) and microfluidic processing in a T-junction device are still under development to prepare MBs with a controlled size [29,32].

MBs can undergo stable oscillation, disruption, fragmentation and coalescence, or a combination of these, depending on the ultrasound exposure conditions. But, the most relevant biophysical effect of ultrasound on MB to enhance gene delivery is cavitation [33,34]. Cavitation can be subdivided into two categories: stable and inertial cavitation. Stable cavitation occurs when MB oscillates stably around a resonant diameter in a low-intensity acoustic field. This generates local shear forces and acoustic microstreaming [34]. At high pressure amplitudes, MB undergo large size variations which make them implode in an event termed as inertial cavitation [35]. MBs collapse violently producing water jetting, shock waves and other inertial phenomena [30]. Both stable and transient cavitations may induce membrane permeabilization [36].

## Mechanisms of sonoporation-induced delivery

**Figure 1:**
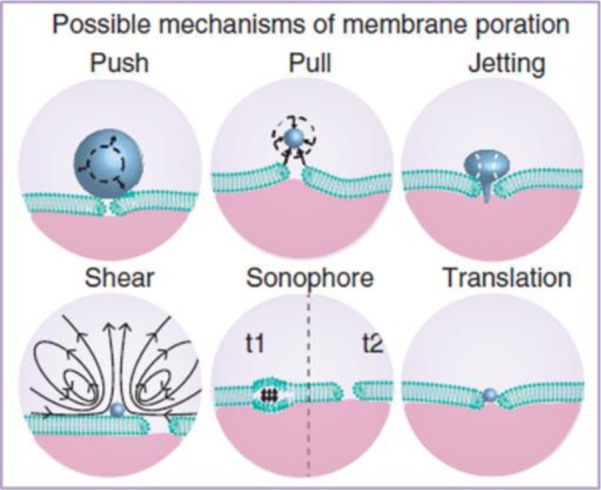
Six proposed mechanisms for pore formation in the plasma membrane induced by sonoporation Reproduced with permission from Delalande et al. [31].

It is still unclear how cells that are subjected to ultrasound and MBs internalize therapeutic compounds, and all cellular responses that are induced in consequence [26,27]. There are five non-exclusive processes for explaining the sonoporation phenomenon: push/pull, jetting, shear and MB translation (Figure 1). It has been hypothesized that expanding MBs might push the cell membrane inward, and that collapsing MB might pull cell membranes outward [37]. These mechanisms require the presence of MB in the close vicinity of cell membranes. The third mechanism is called jetting. A microjet is a fluid protrusion towards a surface during inertial bubble collapse and it only occurs when high acoustic pressures are applied [38]. It is very effective in puncturing cell membranes but there is not yet any proof of cell survival after jetting. Another possibility of pore formation mechanism is the rupture of the cell membrane by the streaming flow created by oscillating MB. The fifth hypothesis states that cavitation nuclei inside cell membranes called sonophores induce membrane swelling during the expansion phase of ultrasound and the deformation could lead to the formation of a pore [39]. Finally, it has been speculated that lipid-encapsulated MBs in compressed phase could pass through cell membranes or channels in the cell membrane, which they had formed. In the case of therapeutic loading, the cargo would be delivered directly into the target cell [40,41].

Experimental observations suggest that pores last as long as vibrating MBs and ultrasound field are present. This is due to the fact that membrane permeabilization is reduced when the ultrasound field is switched off. The pores reseal quickly (after 10 s maximum) once the ultrasound is stopped [42]. We have reported that sonoporation can induce an outward transport of eGFP stably expressed in HeLa cells [43]. The membrane destabilization induced a very short transient release of eGFP from the cytosol of those cells while preserving their viability. This was in line with the recent report on MB oscillations recorded in live with high-speed fluorescence imaging. The videos show that the plasma membrane perforation occurred in 10 s after sonication with a resealing 10 s later [44].

Recently, biophysical insight into the sonoporation mechanism has been reported [45]. A sophisticated set-up comprising an ultrafast bright-field imaging and slow speed epifluorescence microscopy was used to follow the sonoporation of human vascular endothelial cells (HUVECs). Data indicate clearly a correlation between the microsecond-scale MB oscillations and the diffusion of molecules with a second to minute scale. It seems that MBs under US field behave as actuators focusing the energy and inducing local shear stress magnitudes in the plasma membrane, which result in pore formation. With a 3D real-time resonant scanning confocal microscopy, it was observed that the membrane poration started at the point of MB–cell interaction and over a wide area after US application. In addition, the generated pores did coalesce to form a large perforation, which seems not to affect the cell viability. But, this evaluation has to be done at least 2 days post sonoporation to really validate the safety of such events. Moreover, it was observed that a dynamic membrane gap between adjacent cells occurred minutes upon US delivery and it subsisted up to 10 min. This could be the mechanistic explanation of the transendothelial delivery mediated by US and MB proposed to enhance the blood–brain barrier permeability [46].

Despite new knowledge gained the last years, it is still not very clear whether the type of mechanism(s) involved in sonoporation could be both dependent on the MB chemical composition and on the type of tissue [34,37]. Concerning the improved uptake by sonoporation, endocytosis has been also reported to be involved in addition to the uptake via formed pores. Both Hauser et al. [47] and Meijering et al. [48] have demonstrated that when treated with low-intensity ultrasound in presence of MB, cultured cells have an increased endocytosis activity. By using high-speed imaging coupled with confocal microscopy imaging, we showed that Micromarker™ MBs could be pushed into a human carcinoma HeLa cells during sonoporation process under a specific ultrasound setting [40]. This observation has been confirmed by others using real-time imaging as well during ultrasound exposure [49]. They recorded different interactions of MBs composed of DPPC and DSPE-PEG with human melanoma cells with varying acoustic pressure. Low acoustic pressure enhanced the uptake by endocytosis stimulation while high acoustic pressures result in pore formation of the plasma membrane. The primary radiation force seemed to propel the MB towards cell at high velocities inducing pore formation and likely MB translation [41,49]. Nevertheless, we have found that the efficacy of gene delivery was correlated with acoustic parameters conditions where MB translation inside the cell occurred [41]. Interestingly, Hussein et al. [50] showed that ultrasound and MBs could induce a destabilization of early/recycling endosomes allowing a faster release of endosomes content in retinal pigmented epithelial cells. Again, it is not known if such event is valid for different cell types and acoustic conditions.

How cells are recovering from the membrane destabilization and pore formation is an important question, that is worth to be addressed. Leow et al. [51] have performed an elegant study following the recovery process that occurs upon sonoporation. They managed to create a single-pulse ultrasound exposure (1 MHz, 30 cycles, 0.45 MPa of negative pressure) triggering an inertial cavitation of a single anti-VEGF antibody targeted-MB bound to ZR-75-30 breast carcinoma cells. Membrane dynamics were followed in real time after sonoporation and it was observed that membrane blebbing could be a possible action of sonoporated cells to restore plasma membrane integrity. A positive correlation between the pre-exposure MB diameter and the volume of the membrane bleb at the sonoporation site was deduced, thanks to statistical analysis. The initiation of membrane blebbing seems to be more initiated by larger MB size. The blebbing probability for a MB having a size ranging from 1.0 to 1.5 µm is 1/10 cells, while for MB of larger than 3.0 µm, almost all cells exhibited membrane blebbing. Calcium influx is one of the known bioeffects of sonoporation [52]. By following the calcium dynamic, these authors found that intracellular calcium filled the bleb volume. The calcium level inside the bleb and that of cytoplasm returned to the baseline at the same time, over 3 min. This result motivated the authors to postulate that the blebbing could be a physical buffer zone that allows the cell to accommodate the calcium influx resulted by sonoporation. It has to be pointed out that the study has been done only in one type of cell and targeted MB which is bound tightly to the plasma membrane. Again, no one could affirm that blebbing is the unique scenario used by cells to heal the wounded plasma membrane. Cells could also launch more discrete events to strive against the membrane disruption. A lack of mechanistic and deterministic knowledge hinders the rational determination of sonoporation parameters and other factors in order to ensure an efficient and consistent outcome of this method [53].

## MBs as carriers for genes

Ultrasound-enhanced gene delivery has been successfully demonstrated both *in vitro* and *in vivo* [34,54]. The high number of publications relative to the use of ultrasound and MBs for non-viral gene delivery gives evidences about the potentiality of this physical method. The efficacy is dependent on acoustic parameters, the presence and the type of MBs, and the local concentration of the plasmid DNA (pDNA). Commercial ultrasound contrast agents as well as customized lipid- or polymer-shelled MBs had been used in those studies. They were mainly anionic or neutral at pH 7.4 and were not able to interact with pDNA. Therefore, in those studies pDNA was free and thus prone to degradation. As a consequence, the level of gene expression was not as much as expected even though it was at least one or two orders of magnitude higher than that obtained with pDNA alone. In addition, a high amount of pDNA was required. Ultrasound-enhanced gene transfer has been also successful *in vivo*, both with reporters and therapeutic genes. Biological effects and high spatial targeting of transgene expression were achieved, but again, not as high as that obtained with electroporation for instance [55]. Nevertheless, the localized gene expression at the site of ultrasound application as well as the low toxicity observed upon local or systemic administration demonstrates the potentiality of this non-viral gene delivery method. The majority of *in vivo* studies was performed with ultrasound operating at a frequency between 1 and 3 MHz and various types of MBs made with gaseous liposomes or polymers [56]. They have been performed to transfect various organs [31] and MBs used were mostly dedicated to US imaging (Definity™, SonoVue™ or Micromarker™). Rare are studies that report a long-lasting gene transfer. We have found that an efficient and sustained gene transfer up to 100 days in Achilles tendons was possible by using BR14 lipid-shelled MB [57]. BR14 MBs (Bracco Research ®) are composed of a perfluorobutane (C_4_F_10_) gas encapsulated by a phospholipid shell and have a median diameter of 2.3–2.9 μm. Optimal gene transfer was obtained with 1 MHz ultrasound frequency, 200 kPa and 40% duty cycle during 10 min in the presence of 10 µg pDNA and 10 µl BR14 MBs. The gene expression was 100-fold more efficient than with naked pDNA alone. This sustained transgene expression was attributed to an episomal expression of the transgene as deduced by plasmid rescue assay data [57]. As any cells with a low proliferative index, tenocytes may retain introduced pDNA for a long time. It is worth pointing out that this long-lasting gene transfer efficiency (~100 days) is comparable with that obtained in tendons transduced with adenoviral vectors (75 days). Moreover, it is also higher than electroporation method which at optimal electric conditions (200 V for 10 ms or 1200 V for 100 μs) without cytotoxicity, only a modest improvement (two-fold) was obtained compared with free pDNA [58].

Several groups have reported that ultrasound application in the presence of commercial MBs can significantly enhance (more than 200-fold) gene transfer mediated by pDNA complexed with cationic lipids or polyethyleneimine (PEI) [59,60]. In this case, MBs are supposed to act on the plasma membrane to augment pDNA complexes cell uptake.

Since, the oscillation of MB on the plasma membrane is assumed to favour the uptake of pDNA by the cells. Different approaches to bind or load pDNA to MB have been proposed. Complexes of pDNA by either polymer or cationic liposomes were linked to preformed MBs via biotin–streptavidin linkage [61,62]. One issue of this strategy is the large size of the particle that could limit *in vivo* delivery.

Combining MBs with adeno-associated viruses [63,64] or other DNA-condensing transfection reagents such as cationic polymers [65] also offers the possibility to improve expression levels and tissue selectivity. More complex molecular architecture of liposome-based MBs have been also described: (i) bubbles liposomes consisting of PEG-modified liposomes that encapsulate perfluoropropane gas enclosed in PEG–lipid micelles [66], (ii) a hybrid particle made with biotinylated MB attached to small unilamellar biotinylated liposomes made with NBD-cholesterol via avidin molecule (Kheirolomoom et al. [67]) and (iii) layer-by-layer assembly of DNA and poly-l-lysine cationic polymer on a cationic MB [68].

One of the main advantages of using ultrasound as trigger is the controllable spatiotemporal delivery at the insonified tissue area. This targeting could be improved by exploiting molecular targeting strategies to arm MBs with specific cell ligands. The most significant approach consists of MB targeted with a specific antibody. Those MBs can interact and bind with a specific antigen present on a cell membrane even under flow. In this way, molecular targeting would allow MBs to preferentially accumulate in areas of interest and enhance the effects of sonoporation such as VEGFR2-targeted MBs [69] or EGFR-targeted MBs [70] for cancer therapy.

## Cationic MBs for gene delivery

Instead of coupling MBs with pDNA compacted with cationic vectors, one more straightforward strategy is to make cationic MBs. They must bear positively charged lipids or polymers at their shell. The negatively charged DNA–phosphate backbone can interact with positive charges of the shell to form electrostatic complexes [23,71]. In most cases, simple mixing can generate MB–plasmid hybrid vectors. As for other DNA complexes, pDNA compacted on to the surface of cationic MBs are protected against nuclease degradation [23,71], improving its delivery at the insonation site.

**Figure 2:**
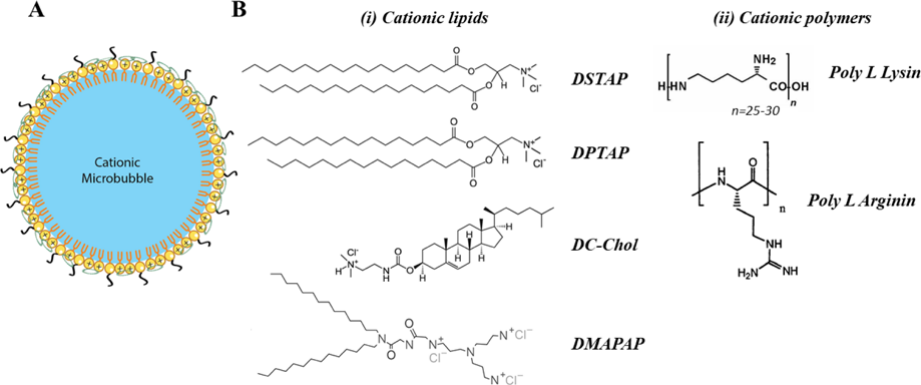
Schematic representation of cationic microbubbles and the most used cationic agents (**A**) Schematic representation of cationic lipid based MBs. Cationic lipids (+) are mixed with PEGylated phospholipids (black tail). Anionic pDNA (green circle) is loaded on MB surface by electrostatic interaction. (**B**) Chemical structure of cationic agents used in MBs formulation. Both cationic lipids (i) and polymers (ii) could be used in MBs formulation. All shown molecules are commercially available, except 2-(3-[bis(3-aminopropyl)amino]propylamino)-N-ditetradecyl-carbamoyl methyl acetamide (DMAPAP).

Table 1 summarizes few studies reporting the use of cationic MB (Figure 2A) that directly complexes pDNA, [71,72], and the chemical structure of mostly used cationic agents are shown in Figure 2B. Gas-filled cationic liposomes were mostly made with the neutral phospholipid 1,2-distearoyl-*sn*-glycero-3-phosphocholine (DSPC) and cationic lipids such as 1,2-distearoyl-3-trimethylammoniumpropane (DSTAP). However, despite a good acoustic response of these MBs, the level of gene transfer was rather low likely due the large size of the resulting particles (>1 µm) and the intracellular fate of pDNA. Cationic MBs targeted CD105 [73] or ICAM-1 [74] protein have been developed for gene delivery of endostatin or angiopoietin-1 respectively.

**Table 1:** Selection of cationic MB reports for pDNA delivery

Cationic lipid	Formulation	ζ potential	Gas	Tissue/Cell targeted	Transfected gene	References
**DMAPAP**	DMAPAP: DMAPAP-PEG2000	+70 mV	C_4_F_10_	Liver	Luciferase (CMV promoter)	[76,77]
**DC-Chol**	DSPC: DSPE-PEG-2000: DPPA:DC-Chol	+22 mV	C_3_F_8_	Infarcted heart	Endostatin	[73,74]
	(0.39: 0.01:0.2:0.2) molar ratio				Angiopoietin-1	
	Combined with ICAM or CD105 antibody				(CMV promoter)	
**DPTAP**	DPPC: DSPE-PEG2000: DSPE-PEG200-biotin: DPTAP	+36.7 mV	C_3_F_8_	C6 brain tumor	Thymidine kinase	[78]
	(9:1:1:1) weight ratio combined with VEGFR2 antibody					
**DSTAP**	DSPC: DPPE-PEG5000: PA: DSTAP (0.59:0.01:0.2:0.2) molar ratio	+15.8 mV	C_4_F_10_	SVR angiosarcoma xenograft	Luciferase (CMV promoter)	[79,23]
**DSTAP**	DSPC: DSTAP:PEG40 (0.43:0.02:0.49)	N/A	C_4_F_10_	HEK293 cells	GFP	[80]
**DSTAP**	DSPC: DSTAP:PEG40 (2 :0.4 :2 weight ratio)	+60 mV	C_4_F_10_	Skeletal muscle	Luciferase (CMV promoter)	[71]
**DSTAP**	DSPC: DSTAP:PEG40 (2:0.4:1 weight ratio)	N/A	C_4_F_10_	Skeletal muscle	VEGF	[81]
**DSTAP**	DSPC: DSTAP:PEG40 (0.43:0.08:0.49 molar ratio)	+4 mV	C_4_F_10_	Skeletal muscle	Luciferase	[82]
**Cationic polymer**	**Formulation**	**ζ potential**	**Gas**	**Organ targeted**	**Transfected gene**	**References**
**Poly-l-arginine**	DSPG: DSPC:DSPE-PEG (7:2:1 molar ratio)	N/A	C_3_F_8_	Kidney, liver, spleen	Luciferase	[65]
**Poly-l-lysine**	DSPG: DSPC:DSPE-PEG (7:2:1 molar ratio)	N/A	C_3_F_8_	Kidney, liver, spleen	Luciferase	[65]

This table is divided in two sections, one where the cationic agent is a lipid and the second where the cationic agent is a polymer. When available, the ζ potential is mentioned. These values cannot be directly compared. For more details regarding the experimental conditions in which these values are being taken, please consult the indicated references.

Abbreviation: DMAPAP, 2-(3-[bis(3-aminopropyl)amino]propylamino)-N-ditetradecyl-carbamoyl methyl acetamide; N/A, non-available.

Other complex MBs have been produced by combining cationic polymer (PEI, polyallanine, poly-l-lysine), cationic peptides as cell penetrating peptide or lipoplexes comprising cationic liposomes with nucleic acids (Table 1). The latter required the use of micelles or avidin/biotin linkage [75]. Despite being interesting, one of the drawbacks of these formulations is their size and the softness that could limit the diffusion.

The global charge of reported cationic MBs is between +15 and +40 mV. The highest one of +60 mV was made with DSTAP:DSPC:PEG40 formulation and used for gene delivery in skeletal muscles [71]. However, as these values have been measured in different experimental setups, they are only indicative and need to be carefully compared.

Several parameters including their acoustic properties, size and stability, and their interaction with nucleic acids need to be deeply investigated to increase the cationic MB effectiveness.

**Figure 3:**
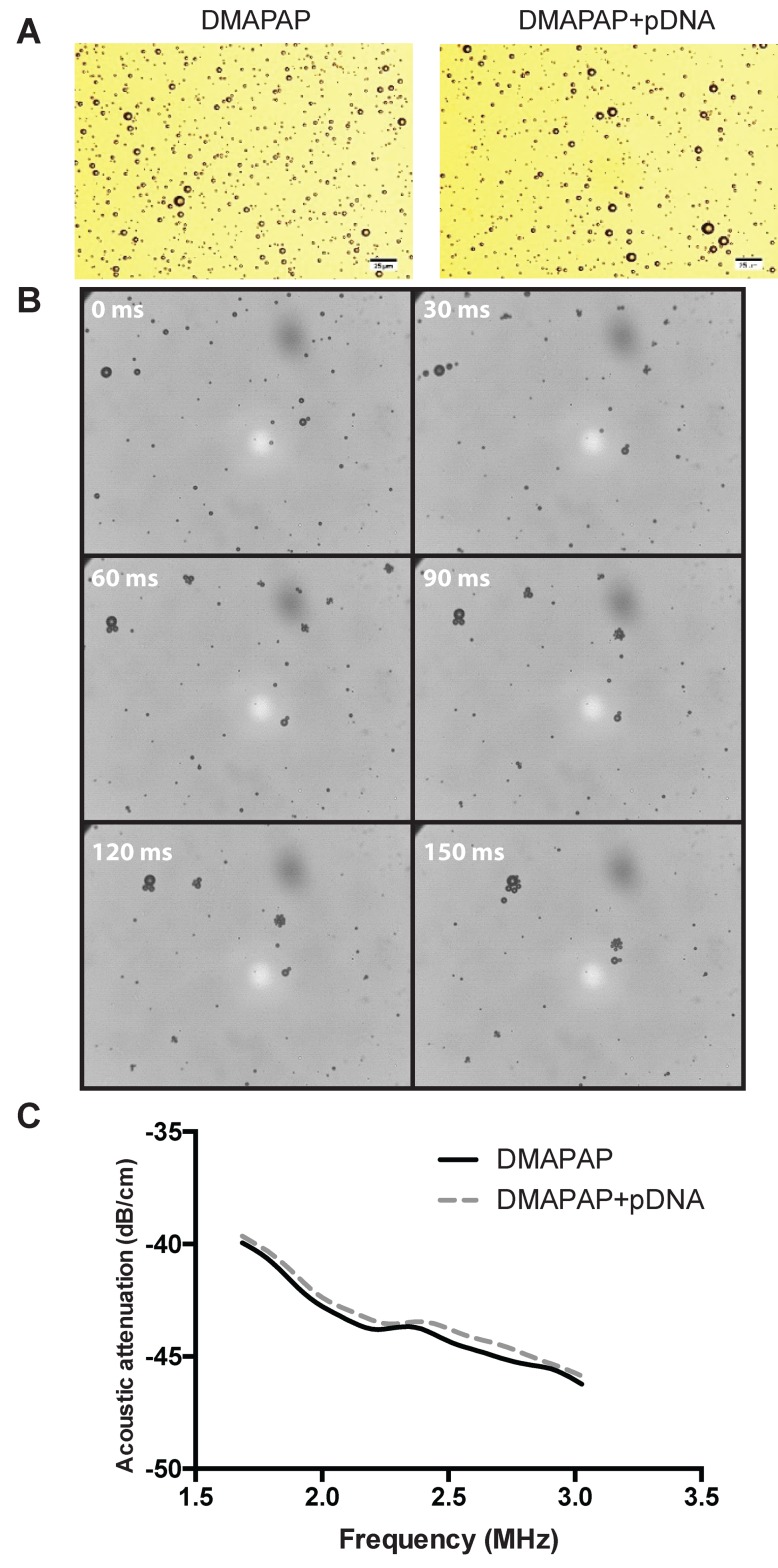
MB acoustic activity and impact of pDNA interaction (**A**) MBs were observed by optical microscopy before and after complexation with pDNA. (**B**) Acoustic activity of 10 µl of DMAPAP MB complexing with 1 µg of pDNA diluted in 10 ml Opticell chamber, the pictures were taken by high-speed imaging during US stimulation (1 MHz, 37 kPa, 40% DC, 10 kHz PRF). (**C**) Impact of DMAPAP MB complexation with pDNA on acoustic attenuation. The acoustic attenuation induced by MBs as a function of ultrasound frequency was measured by using 10 µl of DMAPAP MB complexing with 1 µg of pDNA. The solution was dispersed in 1 ml of water in an ultrasound transparent microcuvette under slow stirring. The microcuvette is positioned in between two 2.25 MHz ultrasound transducers. Ultrasound signal was modulated using a pulser/receiver. The signal was recorded by a numeric oscilloscope and analysed by subtracting the background.

MB stability is one of the main important parameters for efficient gene delivery *in vivo* [83]. Since MB is composed of a gas stabilized by a shell, its surface tension must be low for a better stability [84]. The use of surfactants will decrease surface tension and increase MB stability. The shell will modify linear [85] and non-linear [86] propagation of ultrasound. The prediction of MB oscillations has been widely studied on the basis of the Rayleigh–Plesset equation as a function of MB parameters and the MB shell integrated [87–89]. These models were used to describe the effects of MB composition on its behaviour under ultrasound. Many parameters were taken into account such as the pressure inside MB, surface tension, shell thickness, gas diffusion and MB size. The dynamic behaviour of cationic MB complexing pDNA has never been computed. As an anionic molecule, pDNA interaction on the cationic MB’s shell should modify both the MB’s surface tension as well as MB resonance frequency. Recently, we produced cationic MB with 2-(3-[bis(3-aminopropyl)amino]propylamino)-N-ditetradecyl-carbamoyl methyl acetamide (DMAPAP), a triply charged cationic lipid, that has been shown to interact in pDNA and deliver gene when formulated as liposomes [90]. We analysed the impact of pDNA interaction on the MB morphology and properties. There was no alteration of their size 5 min after complexation with pDNA as observed by optical microscopy (Figure 3A). DMAPAP MB acoustic activity was recorded using high-speed imaging (Figure 3B). It appears that DMAPAP MB complexing pDNA are behaving similarly as anionic MBs in an ultrasound field forming MB clusters that aggregates (30000 fps, 37 kPa, 40% DC, 10 kHz PRF) [91]. MB complexed with pDNA exhibited similar attenuation curves indicating the absence of alteration of their acoustic feature following pDNA interaction. Interestingly, the addition of pDNA to DMAPAP MB did not significantly change the attenuation of the ultrasound signal (Figure 3C).

Those cationic MBs were able to induce a contrast ultrasound imaging signal making them suitable for theranostic applications. The advantage of such system that gives diagnostic information in addition to delivery of therapeutics is well admitted.

**Figure 4:**
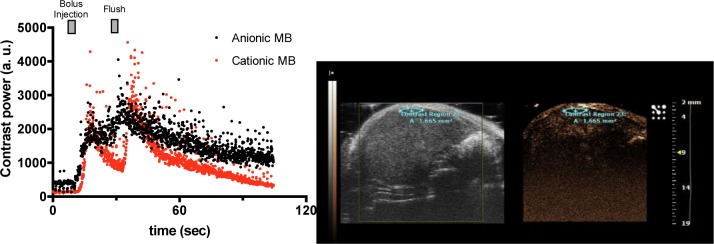
Influence of the MB global charge on contrast imaging Formulations of DSPC MBs with (red curve) or without (black curve) cationic lipids were injected in the mouse tail vein by bolus followed by a flush with saline. Contrast power was analysed in the contrast region drawn on the right-hand side picture.

Nevertheless, these MBs are less stable in blood circulation because of their possible interaction with leucocytes due to their cationic global charge [92]. The addition of a significant amount of cationic lipid (45% molar ratio) in MB formulation comprising DSPC and DSPE-PEG2000 increased their washing out rate in the liver of mice compared with DSPC and DSPE-PEG2000 MB formulation (Figure 4). The cationic MBs diffusion could also be affected by their way of administration. As notified by Christiansen et al. [71], micropores were mainly observed in insonified skeletal muscles after IA injection but not after IV, correlated by an increased efficacy of transfection. The authors attribute this effect to a better perfusion, however, one can hypothesize that IA injection is more efficient because MBs were not subjected to pulmonary filtering leading to MB size sorting. Parameters such as size or stability are able to drive a better tissue perfusion and thus, appear as key parameters in transfection efficiency.

**Figure 5:**
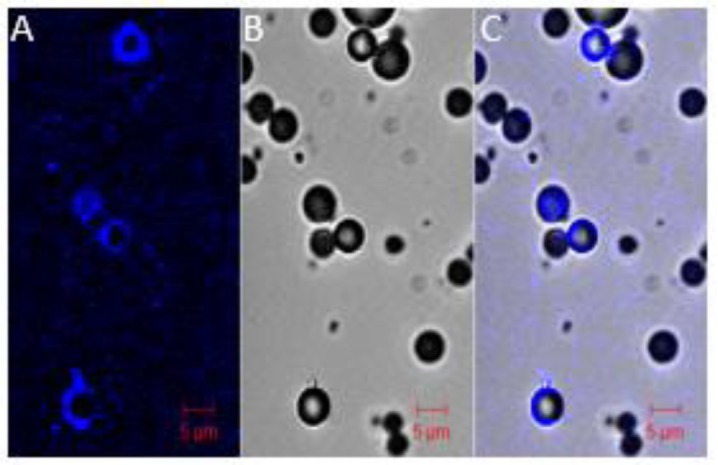
Distribution of pDNA on MB shell Nucleic acids were labelled with Cy5 probe using Mirus kit, 10 µl of DMAPAP MBs were then complexed to 1 µg of pDNA. Confocal microscopy images were taken 5 min after complexation to observe pDNA–MBs complexes. The fluorescence was only found on the MBs shell. (**A**) Cy5-pDNA, (**B**) bright field and (**C**) merge channel.

Besides the classical electrophoresis gel shift assay, confocal microscopy and flow cytometry techniques are useful to evidence the complexation between MB and pDNA. As shown in Figure 5, fluorescent-labelled pDNA can be easily visualized upon binding to the shell of the MB.

**Figure 6:**
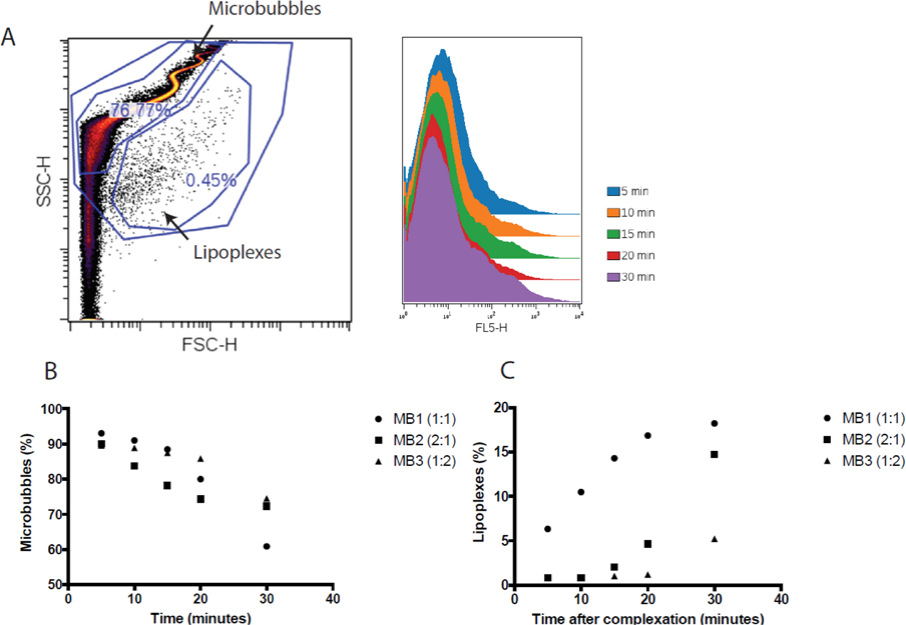
Analysis of cationic MB stability after interaction with pDNA by flow cytometry MBs were prepared with a mixture of cationic lipids, pH-sensitive lipids in a molar ratio of 1:1 (MB1), 2:1 (MB2) or 1:2 (MB3) respectively and 5% of PEGylated phospholipids (DSPE-PEG5000). A complexation between 10 µl of cationic MBs and 1 µg of Cy5-labelled pDNA was made in 10 mM HEPES buffer. (**A**) The FSC-SSC plot of the MBs presents two different populations; MBs and lipoplexes. The MB population was gated and showed pDNA fluorescence in FL5 channel. The proportion of these MBs and lipoplexes populations have been analysed over time of complexation (**B**) MBs and (**C**) for lipoplexes.

When analysed by flow cytometry, MBs exhibit a specific serpentine pattern in the forward-scatter (FSC) compared with side-scatter (SSC) dot plots as previously described [93] (Figure 6A). Flow cytometry analysis offers the possibility to evaluate the stability of MB/pDNA complexes as well. When the MB is destabilized, a new population of smaller particles appeared on the FSC-SSC dot plot. Those dots correspond to pDNA complexed with cationic lipids (lipoplexes) that are formed once the gas inside MB was released. MB associated with pDNA was stable at least up to 30 min. The decreased proportion of entire MB was proportional to the increase in lipoplexes population (Figure 6B,C).

**Figure 7:**
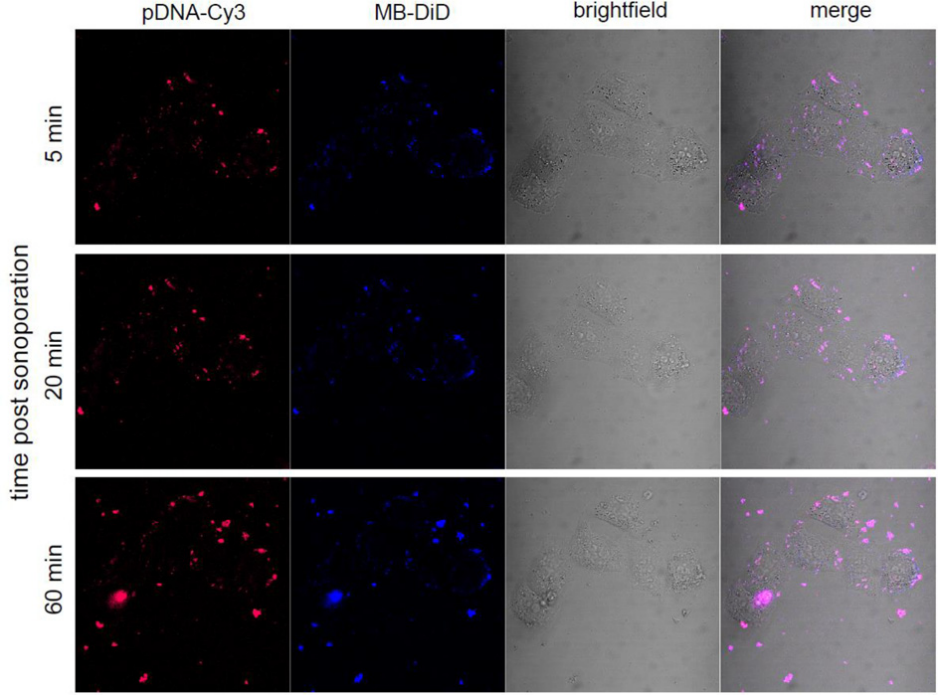
MB–pDNA complexes’ interaction with cells after ultrasound Complexes formed between Cy3-labelled pDNA and DiD-labelled DMAPAP MBs were sonoporated to HeLa cells with the following parameters: 1MHz, 150 kPa, 40% of duty cycle for 1 min. The same cells were then observed by confocal microscopy in live conditions at 5, 20 min and 1 h post sonoporation.

The intracellular processing of cationic MBs complexed with pDNA was monitored by real-time confocal microscopy after sonoporation. For that, the distribution of DiD-labelled DMAPAP MB complexed with Cy3-labelled pDNA 5, 20 and 60 min following 60-s ultrasound stimulation (150 kPa, 20% DC, 10 kHz PRF, 1 MHz), indicated that no dissociation was observed between MB and pDNA (Figure 7). At these time points, there is no evidence that the MB integrity is preserved and there is a high probability that gas dissolved leaving the shell at the point of impact in the cell. The dissociation of the pDNA and the MB lipids must occur at later time allowing pDNA expression. pDNA should then follow classical intracellular lipoplexes pathways through endocytosis and endosomal escape [94]. Since at those acoustic parameters, 16% of cells have been transfected when pDNA-expressing eGFP was used (Figure 10), it also suggests that the amount of pDNA released in the cytoplasm is not as much as expected. Nevertheless, this efficiency is quite good if we take into account the insonation time, which is only 60 s.

**Figure 8:**
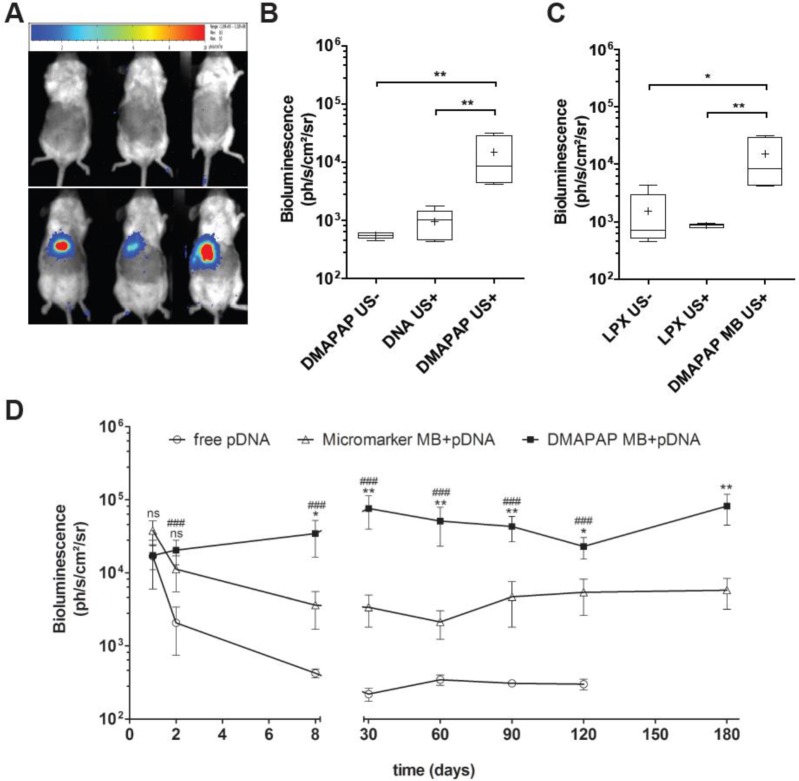
*In vivo* targeted gene delivery in the liver using DMAPAP cationic MBs (**A**) Bioluminescence images obtained 2 days post-injection of DMAPAP lipoplexes (top) and DMAPAP MBs (bottom) both followed by similar US application. (**B**) Bioluminescence signal quantification 2 days after sonoporation when treated with DMAPAP MBs only, DNA and ultrasound or DMAPAP complexing luciferase-encoding pDNA coupled to ultrasound. (**C**) Bioluminescence signal quantification 2 days after treatment with lipoplexes made with DMAPAP lipid and luciferase-encoding pDNA with or without ultrasound compared with DMAPAP MBs. (**D**) Bioluminescence signal kinetics following sonoporation, measurements were done each week up to 180 days post-sonoporation. Values are means ± standard errors, n=9, # represent statistics for pDNA vs DMAPAP MB+pDNA and * for DMAPAP MB+pDNA vs MicroMarker™ MB+pDNA; one symbol p- value<0.05, two symbols p-value<0.01, three symbols p-value<0.001.

DMAPAP MBs were used to deliver luciferase-encoding pDNA in the liver of mice upon ultrasound application. When MB carrying pDNA were administered by bolus injection in the mice tail vein and ultrasound (1 MHz, 930 kPa, 20% DC, 100 Hz PRF during 60 s) turned on in 10 s targeting the liver area, the luciferase expression was restricted to the liver (Figure 8A,B). In contrast, no luciferase expression was recorded when lipoplexes made of pDNA and DMAPAP liposomes were injected without or with US application. Similarly, the injection of naked pDNA without MB and under US pressure did not result in luciferase gene expression in the mice liver. The main result is the stable gene expression obtained with DMAPAP MB/pDNA complexes (Figure 8D). This expression was attributed to hepatocytes transfection rather than endothelial cells as validated by the immunostaining of CD31 [76]. This is in contrast with results obtained with the anionic MB Micromarker™ leading to a similar gene expression in the liver up to 2 days, but which decreased from day 8 (Figure 8D). Using cationic MBs, by stabilizing nucleic acids, allows thus to increase long-lasting gene expression, as shown by studies comparing neutral and cationic MBs for the reporter gene expression [23,79,82]. More interestingly, some studies highlight the interest of cationic MBs for delivery of therapeutic genes. For instead, Leong-Poi et al. [81] demonstrated the positive impact of VEGF transfection in skeletal muscle. These examples are listed in more details in Table 1.

## DMAPAP MB: a common platform for DNA, mRNA and siRNA transfection

Several studies reported the use of cationic liposome bearing MB for efficient transfection of miRNA [95], mRNA [96] and siRNA [97] by sonoporation. These formulations appear more complicated for a generic use for all nucleic acids. Here, we present nucleic acid delivery results obtained using a single cationic MB formulation made with DMAPAP lipid.

**Figure 9:**
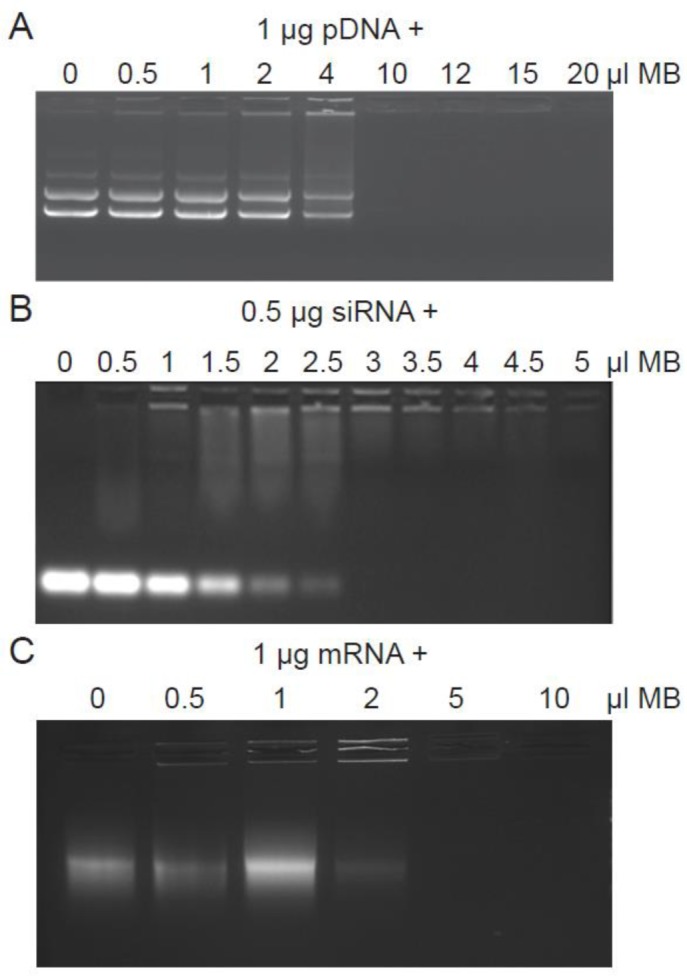
Determination of MBs-nucleic acids ratio Nucleic acids were complexed to an increasing amount of DMAPAP MBs. MBs were complexed with 1 μg of pDNA (A) or 0.5 μg of siRNA (B) or 1 μg of mRNA (C). Agarose gel electrophoresis was performed to evaluate the electrophoretic mobility of different complexes of nucleic acid. While free nuclei migrate (following the control without MBs), complexes are retained in wells due to their big size.

The ability of DMAPAP MB to interact with pDNA, mRNA and siRNA was checked by gel retardation assay. A complete retardation of pDNA migration was observed when 1 µg was associated with 8 µl of DMAPAP MB (Figure 9A). Concerning their interaction with 1 µg mRNA, a complete complexation occurred when 5 µl of MB was used (Figure 9B,C). The same volume was also sufficient to completely complex 0.5 µg of siRNA. As control, no retardation of pDNA was observed with the anionic Micromarker™ MB whatever the amount of MB used.

**Figure 10:**
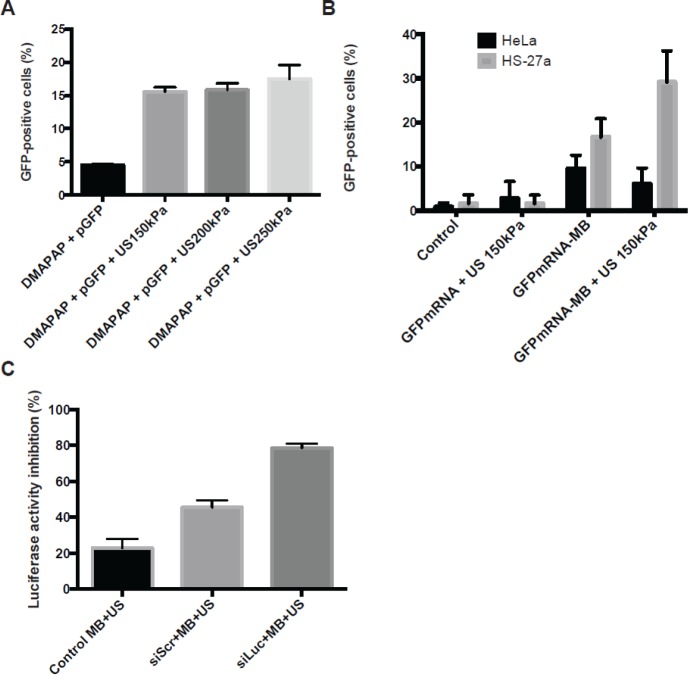
Efficiency of DMAPAP MBs for nucleic acid delivery by sonoporation The percentage of GFP-positive cells was analysed 24 h after sonoporation using (**A**) 1 µg of pDNA encoding GFP complexed with 10 µl of DMAPAP MBs on HeLa cells or (**B**) 1 µg of mRNA encoding GFP complexed with 10 µl of DMAPAP MBs on HeLa (black) and HS-27a cells (grey). (**C**) Percentage of inhibition of luciferase expression after sonoporation using 100 nM of siLuc compared with siScr complexed with DMAPAP MBs on 4T1-Luc cells. For all experiments, 1 MHz ultrasound was used with a duty cycle of 40% a pulse repetition frequency of 10 kHz and a negative peak acoustic pressure of 150 kPa during 60 s.

Figure 10 presents the sonoporation efficiencies of pDNA, mRNA and siRNA. Twenty four hours post sonoporation with pDNA encoding the *eGFP* gene performed during 60 s ultrasound stimulation (150–250 kPa, 1 MHz, 40% DC, 10 kHz PRF), 16% of HeLa cells expressed eGFP (Figure 10A). Under the same conditions, sonoporation with *in vitro* transcribed mRNA-encoding eGFP, HeLa cells did not significantly express eGFP while 30% of HS-27a cells were eGFP positive (Figure 10B). As recently reported, sonoporation is also dependent on the cell type [98]. Regarding transfection of HeLa cells, the all-cell population seemed to express GFP even though the amount of GFP was low compared with pDNA sonoporation (not shown). This was explained by the difficulty of pDNA to reach the nucleus. In contrast, the mRNA translation requires its delivery in the cytosol. Concerning siRNA delivery, the sonoporation efficiency was evaluated on a 4T1-Luc cell line stably expressing the luciferase gene. The sonoporation was made with 60 s ultrasound stimulation (150 kPa, 1 MHz, 40% DC, 10 kHz PRF) and 100 nM anti-luciferase siRNA (siLuc). Results show that siLuc induced two-fold more inhibition compared with the scramble siRNA (siScr) (Figure 10C). Unfortunately, a high off-target effect was also observed with the siScr as for other delivery methods.

Previous studies estimated that cationic MB could be conjugated with ~20000 plasmids per MB [71,80]. In our study, we used a miniplasmid called pFAR (3750 bp) [99]. According to pFAR size, 1.903 × 10^12^ DNA copies were found to interact with 2 × 10^7^ MB when 10 µl of DMAPAP MB was mixed with 0.5 µg pFAR. Therefore, ~26300 pDNA molecules were conjugated per DMAPAP MB, a value in agreement with that reported by the standard cationic DSTAP MB for example (6700 pDNA molecules per MB) [71]. Thus, with smaller nucleic acids—mRNA and siRNA—same amount of MB could complex more (up to ten-fold) mRNA or siRNA copies. This was in line with the determination of the optimal mRNA/MB or siRNA/MB ratios detected by gel retardation (Figure 8). Nevertheless, even though the nucleic acid/MB ratio differed between pDNA and mRNA, they present the same repartition on the MB shell. Our results demonstrated also that direct interaction of nucleic acids did not affect MB morphology and their acoustic properties. However, the nucleic acids type seemed to influence the MB stability. As analysed by flow cytometry, more than 60% of intact MB was still observed up to 30 min with pDNA while after 5 min, MB with mRNA were rapidly transformed in lipoplexes. Nevertheless, mRNA sonoporation performed within this time lapse was really mediated by mRNA/MB and not by mRNA lipoplexes. In order to improve the pDNA or mRNA sonoporation efficiency, understanding the molecular and cellular mechanism(s) involved in their respective delivery constitutes a crucial step. In this field, recent studies realized with anionic and/or neutral MB, suggested that membrane perforation by MB cavitation promoted the nucleic acid entry by a clathrin-dependent endocytosis pathway [41] and ~30% of free pDNA were cleared by autophagosomes [100]. Accordingly, the development of MB stabilized by a cationic shell could take advantage of cationic lipids already used in lipofection to improve endosome escape and cytosolic delivery of pDNA [101,102]. Today, our knowledge regarding the mRNA or siRNA uptake after cationic MB mediated sonoporation is very scarce and deserves to be investigated. Altogether, our data show that DMAPAP MBs could be a promising new platform for nucleic acids delivery.

## Conclusion and discussion

The main advantages of MB and ultrasound assisted delivery are its non-invasiveness and the controlled location of the delivery inside a specific area. Therefore, it offers an attractive method for non-invasive targeted gene delivery. Reported data from *in vivo* sonoporation delivery indicate that the level and duration of gene expression seemed to be highly dependent on both the formulation and the targeted tissue. ζ potential, nucleic acid/MB ratio, optimal surfactant composition are as many different parameters for better efficiency, stability and tissue perfusion. More knowledge has to be acquired to understand the differences.

In the recent years, scientists from different disciplines were very proficient in acquiring new knowledge about the sonoporation mechanism. The specific set-up made of sonoporation chamber mounted on confocal microscopy and coupled with high-speed camera has allowed us to report the translation of MB inside the cells during sonoporation for the first time [40]. This set-up has inspired many laboratories which in turn have adapted it with ultrafast imaging or other equipments [44]. These studies have permitted to validate hypotheses and highlight novel cellular events as membrane blebbing that occur at the plasma membrane during sonoporation. The observation of neutral MB translation inside cells and the location of MB and pDNA inside the cells have prompted us to produce novel cationic MB. We have chosen to exploit cationic lipids that we developed for lipofection-based gene delivery [90]. Optimal formulation and optimal acoustic parameters were determined, thanks to US imaging. Side by side comparison experiments have deduced the superiority of sonoporation compared with lipofection. Now, it would be worth to investigate the biophysical insight into such cationic MB formulation to choose their use properly.

The efficiency of this delivery system could be highly improved by the possibility of producing cationic MBs that can be targeted with a specific moiety. This is quite precarious since any action on the shell of MB impact on its fate as discussed before. This technology becomes very sophisticated if one would like to arm the cationic MB with targeted ligand or antibody.

Reports from the last 2 years show the importance of understanding MB and nucleic acids interaction as well as MB and cell interactions. It is easy to be convinced about the importance of cellular bioeffects that may depend on the nature of sonoporated cells and the tissue location. Events as stimulation of mechanotransduction leading to cell migration or proliferation might be desirable for regenerative medicine but dangerous for cancer treatment. Mastering those events will allow us to finely tune MB and acoustic parameters dedicated for one purpose.

## Abbreviations

Cy5: cyanine 5
Cy3: cyanine 3
DMAPAP: 2-(3-[bis(3-aminopropyl)amino]propylamino)-N-ditetradecyl-carbamoyl methyl acetamide
DSPC: 1,2-distearoyl-*sn*-glycero-3-phosphocholine
DSTAP: 1,2-distearoyl-3-trimethylammoniumpropane
FSC: forward scatter
IA: itra arterial
IV: intra veinous
MB: microbubble
pDNA: plasmid DNA
PEI: polyethyleneimine
siLuc: luciferase siRNA
siScr: scramble siRNA
SSC: side scatter
US: ultrasound
